# Supplementary material for: Effect of ultrasound-guided transverse abdominal plane block on neutrophil-to-lymphocyte ratio, platelet-to-lymphocyte ratio, and systemic immune inflammation index in patients undergoing radical resection of endometrial carcinoma
Source: PLoS One. 2024 Dec 5;19(12):e0315175. doi: 10.1371/journal.pone.0315175 (PMC11620674; doi:10.1371/journal.pone.0315175)
Supplement: S2 File — (DOCX) [file pone.0315175.s003.docx]

**超声引导TAP阻滞对子宫内膜癌根治术患者的中性粒细胞/血小板与淋巴细胞比率的影响**

**一、研究依据**

**（1）研究意义**

子宫内膜癌是女性生殖系统最常见的恶性肿瘤之一。近年来，随着女性长期服用雌激素、肥胖、高血压及不孕等高危因素的增加，子宫内膜癌的发病率有明显上升的趋势。子宫内膜癌临床症状出现较早，早期患者居多，预后较好。然而一旦发生复发、转移，其预后不佳。子宫内膜癌的发病机制目前尚不确切，可能与无孕激素拮抗的雌激素长期作用下机体产生的慢性炎症和免疫反应有关。而手术是治疗子宫内膜癌最主要的方法，而围手术期是一个多因素决定促进或阻碍残余肿瘤细胞转移的关键窗口期。子宫内膜癌根治术是切除原发肿瘤及转移淋巴结最有效的方法，然而手术过程中一些残余的肿瘤细胞在机体应激或免疫抑制的微环境中通过机械方法、淋巴及血液系统进一步微转移。腹腔镜子宫内膜癌根治术是子宫内膜癌患者的常规治疗方法，虽然手术看似创伤小，操作简单，但对麻醉和镇痛的要求较高，需要有效的麻醉管理，以减轻术后疼痛，促进康复。

围手术期影响癌症复发、转移的因素很多，包括麻醉、免疫系统、输血、低体温、高血糖、术后疼痛等。麻醉对肿瘤增值、迁移和侵袭影响的机制尚不清楚。一些回顾性研究报道了区域麻醉可以降低肿瘤转移和复发的风险。这主要是因为区域麻醉减弱了手术引起的神经内分泌反应，并到达中枢神经系统抑制有害的神经冲动。有部分围手术期选择不同麻醉方法是否会直接或间接影响肿瘤患者预后的回顾性或前瞻性研究，但结论并不统一。恶性肿瘤的复发、转移仍难以控制，这可能与麻醉等多种影响预后的因素密切相关。

传统上，麻醉医师在不确定麻醉药、麻醉方法是否影响肿瘤复发和转移的情况下进行镇静、麻醉和术后镇痛，从而可能影响肿瘤的迁移和复发。随着超声技术的不断优化，超声引导腹横肌平面阻滞(transverse abdominal plane block, TAPB)能够在可视下操作，在临床麻醉研究中受到广泛关注。TAPB能有效阻断前腹壁的神经，减少手术伤害性刺激的传入，降低交感神经兴奋，血流动力学更稳定；同时，TAPB能阻断传入神经冲动，有效避免中枢和外周的疼痛敏化及炎性因子释放，优化了术中、术后镇痛。

超声引导下TAPB作为一种区域麻醉技术，能够通过局部麻醉药的作用有效地对腹内斜肌与腹横肌之间腹壁传入神经纤维产生较强的阻滞作用，从而有效地阻断了腹壁痛觉的传入。超声引导下的TAPB可减少视野盲症，清晰显示神经及周围组织，最大限度减少操作不当引起的并发症发生率，进一步减轻对机体的影响，优化整体麻醉效果。

有研究显示，TAPB能阻滞第8胸椎(T_8_)至第1腰椎(L_1_)神经前皮支，其支配的皮肤和肌肉可至正中线。妇科腹腔镜的前腹壁切口恰由第7胸椎(T_7_)至L_1_，脊神经前支支配，其前皮支行走于腹横肌筋膜层，TAPB恰好为妇科腹腔镜手术提供了术中和术后腹壁的镇痛。亦有研究表明，TAPB也能对内脏痛提供一定的镇痛效果。通过阻断腹壁前神经，TAPB减轻了腹壁区域的疼痛。研究表明，TAPB多用于下腹部手术，能够有效缓解术后疼痛、抑制炎症反应，提高术后免疫功能。研究显示在某些恶性肿瘤中机体的炎症反应先于肿瘤的发展，而机体的炎症有助于恶性肿瘤细胞的增殖、存活及转移。

炎症反应主要表现为外周血象的改变，即中性粒细胞、血小板增多和淋巴细胞减少。一方面，在炎症反应中，中性粒细胞的升高可抑制免疫细胞比如淋巴细胞、活化T细胞及NK细胞，从而抑制免疫系统；而作为机体最重要的免疫细胞，淋巴细胞(T、B细胞)的主要功能是抑制肿瘤的生长、促进肿瘤细胞的凋亡，淋巴细胞减少提示机体的免疫系统功能受抑制。另一方面，由肿瘤细胞、非恶性基质细胞和迁移性造血细胞组成肿瘤生存进展及转移的微环境，而中性粒细胞产生的细胞因子如COX-2、PGE2 和趋化因子影响炎症细胞募集和激活，从而对肿瘤的微环境的平衡产生重要影响。另外，血小板不仅参与机体的凝血功能，还可能分泌某种细胞因子，直接或者间接的参与机体的免疫反应，也是反应机体炎症反应的一项指标。

中性粒细胞在肿瘤微环境中具有促进肿瘤进展的作用，可特异性地支持肿瘤转移起始过程，如释放白三烯、活性氧等，使癌细胞更易于种植转移。中性粒细胞与淋巴细胞比率（neutrophil/lymphocyte ratio, NLR）及血小板与淋巴细胞比率（platelet/lymphocyte ratio, PLR）可以反映机体促肿瘤和抗肿瘤的状态，是多种肿瘤患者术后早期复发和良好预后的独立因素。PLR是同时反应机体凝血系统和免疫状态的指标，与肿瘤的转移复发存在相关性，PLR升高与肿瘤生存期缩短存在相关性。NLR、PLR 是一个反应全身炎症状态和免疫状态的简单有效指标。

外周血炎症细胞如中性粒细胞、淋巴细胞及血小板由于存在个体差异而不稳定，但是NLR及PLR相对稳定，对肿瘤患者预后有较高的预测价值。当NLR和/或PLR比值增高时候，提示机体的炎症及免疫反应受抑制，导致对肿瘤细胞的抑制作用减弱，表明肿瘤患者预后不良。

**近年以来，非特异性炎症及免疫反应成为肿瘤发生、发展的研究热点。然而，TAPB在减少术后疼痛和保护免疫功能方面的研究尚不充分，TAPB影响免疫功能的机制尚不明确。TAPB对子宫内膜癌患者围手术期的NLR、PLR及预后是否有影响尚不明确。**

**（2）国内外研究现状**

研究表明，神经阻滞复合全身麻醉比单纯全身麻醉术后拔管时间快，躁动发生率低，苏醒质量好。研究显示，TAPB组术后76 h免疫功能指标与术前比较无显著差异，但显著高于对照组，提示接受TAPB患者免疫功能受影响较小；TAPB能安全有效地应用于妇科腹腔镜手术，术中血流动力学平稳，术后镇痛效果优良，无不良反应，有利于患者术后早期下地行走和早期康复，缩短术后住院时间；TAPB可增强腹腔镜宫颈癌根治术后患者的镇痛效果，稳定患者体征，对患者免疫功能影响小，安全性高。

TAPB联合全身麻醉能降低外周血中性粒细胞计数及NLR水平，可能与其减轻免疫抑制相关，不同的麻醉药物对免疫、复发和转移有不同的影响。研究表明，大多数静脉全麻药物可抑制免疫系统功能，影响癌症进展。局麻药通过阻断神经细胞膜上的电压门控钠通道(VGSC)发挥作用。肿瘤细胞膜上存在VGSCs，与肿瘤细胞侵袭、转移有关。局部麻醉可增加NK细胞活性，维持Th1/平衡Th2比值，降低术中血浆皮质醇、儿茶酚胺水平。研究表明，局部麻醉可以减少应激反应，保护肿瘤患者的免疫功能，减少阿片类使用。此外，血管内皮生长因子C（VEGF-C）、TGF-β1、酸性成纤维细胞生长因子、基本成纤维细胞生长因子和胎盘生长因子水平在接受异丙酚的患者的静脉中减少，并与椎旁阻滞麻醉结合。这些生长因子促进血管生成和转移性肿瘤的形成，提示麻醉方法影响原发性乳腺癌患者血浆中血管生成相关因子的水平，进而影响肿瘤的复发和转移。与单纯全身麻醉相比，全身麻醉联合硬膜外麻醉可提高临床生存率，改善预后。Short等人的一项多中心随机对照临床研究报道，与全麻联合术后静脉镇痛相比，全麻联合术后硬膜外镇痛既没有降低腹部手术患者的肿瘤复发风险，也没有延长患者的无瘤生存期。

有研究对多种恶性肿瘤进行Meta分析发现，高水平NLR对患者的预后有影响，尤其当NLR> 4.0时，NLR与较差的总体生存率相关。围手术期由于手术应激、炎症和免疫系统抑制均可导致NLR升高，手术应激引起中性粒细胞凋亡的抑制也可能起到作用。多项研究已证实，NLR、PLR的升高与实体肿瘤患者的预后独立相关。王媛秋等研究表明，术前NLR和PLR增高对卵巢癌患者的预后有一定的评估价值，同样的研究结果也在其它实体肿瘤的相关研究中得到证实。研究报道，术前高NLR可作为胃癌分期术后复发的独立因素。有研究显示，术前NLR>3.4是影响患者无病生存期（disease-free survival, DFS）和总生存期(overall survival, OS)的独立危险因素，且NLR高值组分别比NLR低值组DFS和OS低。超声引导下TAPB联合全身麻醉不仅能够为胃癌根治术患者提供良好的术后镇痛，还能降低术后中性粒细胞及NLR水平。Kim等结合NLR的结果提示，TAPB联合全身麻醉相对于单独全身麻醉的优势主要体现在抑制中性粒细胞增多，减轻中性粒细胞对肿瘤微环境的影响。

综上所述，**本课题设想观察超声引导TAPB联合全身麻醉对子宫内膜癌根治术患者外周血中NLR、PLR是否有影响，为其在子宫内膜癌根治术患者中的临床使用提供理论依据。**

**二、主要研究内容**

**（1）观察超声引导下腹横肌平面阻滞（TAPB）对子宫内膜癌根治术患者外周血中性粒细胞与淋巴细胞比率（NLR）、血小板与淋巴细胞比率（PLR）的影响。**

在全身麻醉联合TAPB下进行腹腔镜子宫内膜癌根治术，比较单独全身麻醉组（GA组）和全身麻醉联合TAPB组（GA+TAP组），术前及术后24h、72h NLR及PLR值的差异。探讨超声引导下TAPB对子宫内膜癌根治术患者外周血NLR、PLR是否产生影响。

**（2）观察超声引导下TAPB对子宫内膜癌根治术患者术中静脉麻醉药使用情况、术后镇痛的影响。**

在全身麻醉联合TAPB下进行腹腔镜子宫内膜癌根治术，比较GA组和GA+TAP组术中麻醉药的使用情况和术后疼痛的差异。探讨超声引导下TAPB对子宫内膜癌根治术患者，是否可减少麻醉药量的使用，减少术后恶心、呕吐的发生，减少术后阿片类药物的使用量，改善术后疼痛，减少并发症。

**三、研究方法**

（1）**样本量计算**：根据预实验结果，NLR两组72h后分别为4.80±1.05和5.60±1.30。以α=0.05,1-β=0.8，采用PASS 15.0计算样本量约为每组36例，考虑一定的失访率，在估算的样本量基础上增加20%，每组纳入约45例。

（2）**研究对象:**纳入2023年6月7日~2024年6月1日在石河子大学医学院第一附属医院择期行子宫内膜癌根治术患者90例。

（3）**纳入标准：**①病理确诊为子宫内膜癌；②均拟行腹腔镜子宫内膜癌根治术；③美国麻醉医师协会（ASA）分级Ⅰ~Ⅲ级；④年龄30~65岁；⑤BMI 18~ 30 kg/m2 ；⑥初诊患者，未接受任何系统治疗；⑦未发生其他部位的远处转移；⑧同一组手术医师手术。

（4）**排除标准：**①穿刺部位皮肤感染，合并急慢性感染性疾病者②合并其他恶性肿瘤，神经系统病变，内分泌系统功能障碍及血液系统疾病；③凝血机制异常；④合并严重的重要器官疾病；⑤术前3个月内进行其他重大手术。

**四、研究过程**

（1）**伦理审核及临床试验注册**：本试验经石河子大学第一附属医院伦理委员会批准(KJ2023-015-01)，并在中国临床试验注册中心注册(ChiCTR2300072186)。在任何程序开始之前，必须获得所有参与者或其法定代理人的书面知情同意。

（2）**试验分组：**采用随机数字表法将子宫内膜癌根治术患者各分为两组（每组50例）：TAPB+全身麻醉组（TAP+GA组）和单独全身麻醉组（GA组）。在整个研究过程中，患者、进行数据收集和术后随访的研究人员以及临床工作人员对随机分配不知情。

（3）**试验方法：**患者术前常规禁食8h、禁饮2h，入手术室后开放外周静脉并常规监测心率、心律、无创血压及 SpO_2_。备好急救药品及设备后实施全身麻醉诱导，面罩去氮给氧后依次静脉注射咪达唑仑0.05 mg/kg、丙泊酚2.0 mg/kg、枸橼酸舒芬太尼0.5 μg/kg 和顺阿曲库铵0.2mg/kg，肌肉松弛后在视频喉镜下行经口气管插管，插管成功后行机械通气，根据 P_ET_CO_2_调整呼吸机参数，使P_ET_CO_2_维持在 35~45mmHg。持续静脉输注丙泊酚4~8 mg·kg^‒1^·h^‒1^、瑞 芬 太 尼0.1~0.2μg·kg^‒1^·min^‒1^、顺阿曲库铵1-2μg·kg^‒1^·min^‒1^，维持BIS值在40-60及肌肉松弛状态。围手术期低血压和心动过缓分别给予40 μg去氧肾上腺素和0.5 mg阿托品治疗。TAP+GA 组患者于麻醉诱导后消毒腹部皮肤，使用彩色多普勒超声仪高频线阵探头，探头用腔镜套包裹，将超声探头置于前侧腹壁腋中线与脐水平相交处，获取腹横肌平面内超声图像，根据平面内超声图像将穿刺针引导至腹横肌平面，当针尖到达腹内斜肌与腹横肌之间的筋膜层且回抽无血、无气后注入0.9%氯化钠注射液2 ml，确定位置无误后于腹部两侧分别注入0.375％罗哌卡因20 ml，超声观察药液扩散情况并确定有无并发症。单独GA组未行TAPB。

手术结束时给予单次静脉注射0.1 μg/kg舒芬太尼预先镇痛剂量。所有患者术后均采用常规PCIA进行疼痛管理(在100 mL生理盐水中加入2 μg/kg舒芬太尼、0.06 mg/kg酒石酸丁托啡诺和8 mg昂丹司琼，背景输注2ml / h，每次2 mL，闭锁间隔15分钟)。当患者术后镇痛效果不理想时(定义为静息VAS评分≥4)，建议患者按压镇痛泵。如果连续两次按压后疼痛仍未减轻，主治医生将静脉注射单剂量1mg酒石酸丁托啡诺进行补救性镇痛。

（4）**观察指标**：收集患者一般资料（年龄、BMI、ASA和疾病的临床病理分期）；记录两组患者手术时间、麻醉时间及术中瑞芬太尼用量。于术前及术后24h、72 h检测两组患者外周血中性粒细胞、血小板、淋巴细胞计数及计算NLR、PLR值。记录两组患者术后6h、12h、24h、48 h VAS 疼痛评分、镇痛泵有效按压次数、术后首次下床时间、术后首次排气时间及术后恶心呕吐不良反应的发生情况。

（5）**统计分析方法**：采用SPSS 25.0和GraphPad Prism 9.5.1软件对数据进行分析和可视化。对测量数据的正态性进行评估，对于正态分布的数据，报告平均值±标准差(X±SD)。采用独立样本t检验进行比较分析。对于非正态分布的数据，采用中位数(四分位数范围)[M (Q1, Q3)]，采用Mann-Whitney U检验进行组间比较。分类数据以比例或百分比表示，组间比较采用卡方检验。P值<0.05认为有统计学意义。

Effect of ultrasus-guided TAP block on neutrophil/platelet-lymphocyte ratio in patients undergoing radical endometrial carcinoma surgery

**1 Research basis**

1. **Research significance**

Endometrial cancer is one of the most common malignant tumors of the female reproductive system. In recent years, with the increase of high risk factors such as long-term estrogen use, obesity, high blood pressure and infertility, the incidence of endometrial cancer has a significant rising trend. The clinical symptoms of endometrial cancer appear early, and most patients in the early stage have a good prognosis. However, once recurrence and metastasis occur, the prognosis is poor. The pathogenesis of endometrial cancer is still unclear, and it may be related to the chronic inflammation and immune response produced by the body under the long-term effect of estrogen without progesterone antagonism. Surgery is the most important method for the treatment of endometrial cancer, and the perioperative period is a key window period in which multiple factors determine whether to promote or hinder the metastasis of residual tumor cells. Radical resection of endometrial carcinoma is the most effective way to remove the primary tumor and metastatic lymph nodes. However, during surgery, some residual tumor cells are further micro-metastasized through mechanical methods, lymphatic and blood systems in the microenvironment of body stress or immunosuppression. Laparoscopic radical hysterectomy for endometrial cancer is a routine treatment for patients with endometrial cancer. Although the operation seems to be less traumatic and simple, it requires high anesthesia and analgesia, and effective anesthesia management is needed to reduce postoperative pain and promote rehabilitation.

There are many factors affecting cancer recurrence and metastasis in perioperative period, including anesthesia, immune system, blood transfusion, hypothermia, hyperglycemia, postoperative pain and so on. The mechanism of the effect of anesthesia on tumor proliferation, migration and invasion remains unclear. Several retrospective studies have reported that regional anesthesia can reduce the risk of tumor metastasis and recurrence. This is mainly because regional anesthesia attenuates the neuroendocrine response caused by surgery and reaches the central nervous system to suppress harmful nerve impulses. There are some retrospective or prospective studies on whether different anesthesia methods in perioperative period will directly or indirectly affect the prognosis of tumor patients, but the conclusions are not uniform. The recurrence and metastasis of malignant tumors are still difficult to control, which may be closely related to many prognostic factors such as anesthesia.

Traditionally, anesthesiologists have performed sedation, anesthesia, and postoperative analgesia without being certain whether anesthetics or methods of anesthesia affect tumor recurrence and metastasis, which may affect tumor migration and recurrence. With the continuous optimization of ultrasound technology, ultrasound-guided transverse abdominal plane block (TAPB) can be operated visually and has received widespread attention in clinical anesthesia research. TAPB can effectively block the nerves in the anterior abdominal wall, reduce the afferent injury-induced surgical stimulation, reduce sympathetic nerve excitation, and make hemodynamics more stable. At the same time, TAPB can block afferent nerve impulses, effectively avoid central and peripheral pain sensitization and inflammatory factor release, and optimize intraoperative and postoperative analgesia.

As a regional anesthesia technique under the guidance of ultrasound, TAPB can effectively block the afferent nerve fibers of the abdominal wall between the internal oblique muscle and the transverse muscle of the abdomen through the action of local anesthetic, thus effectively blocking the afferent pain sensation of the abdominal wall. TAPB under ultrasound guidance can reduce visual field blindness, clearly display nerves and surrounding tissues, minimize the incidence of complications caused by improper operation, further reduce the impact on the body, and optimize the overall anesthetic effect.

TAPB has been shown to block the anterior cutaneous branch of the 8th thoracic (T8) to 1st lumbar (L1) nerve, which innervates skin and muscle to the median line. The anterior abdominal incision of gynecologic laparoscopy is innervated by the anterior branch of spinal nerve from the 7th thoracic vertebra (T7) to L1, and its anterior cutaneous branch is located in the fascia layer of transverse abdominal muscle. TAPB exactly provides intraoperative and postoperative analgesia of abdominal wall for gynecologic laparoscopic surgery. Studies have also shown that TAPB can also provide a certain analgesic effect on visceral pain. By blocking the anterior nerves of the abdominal wall, TAPB relieves pain in the abdominal wall area. Studies have shown that TAPB is mostly used in lower abdominal surgery, which can effectively relieve postoperative pain, inhibit inflammatory response, and improve postoperative immune function. Studies have shown that in some malignant tumors, the inflammatory response of the body precedes the development of the tumor, and the inflammation of the body contributes to the proliferation, survival and metastasis of malignant tumor cells.

The inflammatory response was mainly manifested by the changes of peripheral blood images, namely neutrophils, thrombocytopenia and lymphocytopenia. On the one hand, in the inflammatory response, the rise of neutrophils can inhibit immune cells such as lymphocytes, activated T cells and NK cells, thus suppressing the immune system; As the most important immune cells in the body, the main function of lymphocytes (T and B cells) is to inhibit the growth of tumors and promote the apoptosis of tumor cells. The decrease of lymphocytes indicates that the function of the immune system is inhibited. On the other hand, tumor cells, non-malignant stromal cells and migratory hematopoietic cells constitute the microenvironment for tumor survival, progression and metastasis, while neutrophil-produced cytokines such as COX-2, PGE2 and chemokines affect the recruitment and activation of inflammatory cells, thus having an important impact on the balance of tumor microenvironment. In addition, platelets not only participate in the body's clotting function, but also may secrete some cytokines, directly or indirectly participate in the body's immune response, and is also an indicator of the body's inflammatory response.

Neutrophils play a role in promoting tumor progression in the tumor microenvironment, and can specifically support the initiation process of tumor metastasis, such as releasing leukotrienes, reactive oxygen species, etc., so that cancer cells are easier to implant and metastasize. The ratio of neutrophil/lymphocyte ratio (NLR) and platelet/lymphocyte ratio (PLR) can reflect the pro-tumor and anti-tumor status of the body. It is an independent factor for early postoperative recurrence and good prognosis in patients with multiple tumors. PLR is an indicator that reflects the coagulation system and immune status of the body at the same time, which is correlated with the metastasis and recurrence of tumors, and the increase of PLR is correlated with the shortening of tumor survival. NLR and PLR are simple and effective indicators of systemic inflammation and immune status.

Peripheral blood inflammatory cells such as neutrophils, lymphocytes and platelets are unstable due to individual differences, but NLR and PLR are relatively stable, and have high prognostic value for tumor patients. When the ratio of NLR and/or PLR is increased, it indicates that the inflammation and immune response of the body are suppressed, resulting in a weakened inhibitory effect on tumor cells, indicating a poor prognosis of tumor patients.

In recent years, non-specific inflammation and immune response have become the focus of research on tumor occurrence and development. However, the effects of TAPB on reducing postoperative pain and protecting immune function have not been fully studied, and the mechanism of TAPB affecting immune function is still unclear. The effect of TAPB on perioperative NLR, PLR and prognosis of patients with endometrial cancer remains unclear.

1. **Research status at home and abroad**

Studies have shown that the time of extubation after nerve block combined with general anesthesia is faster, the incidence of agitation is lower and the quality of recovery is better than that after general anesthesia alone. The study showed that the immune function indexes of TAPB group at 76 h after surgery had no significant difference compared with those before surgery, but were significantly higher than those of control group, suggesting that the immune function of patients receiving TAPB was less affected. TAPB can be safely and effectively applied in gynecological laparoscopic surgery, with stable intraoperative hemodynamics, excellent postoperative analgesia and no adverse reactions, which is conducive to early postoperative walking and rehabilitation of patients, and shorten postoperative hospitalization time. TAPB can enhance the analgesic effect of patients after radical laparoscopic cervical cancer surgery, stabilize the physical signs of patients, and have little impact on the immune function of patients with high safety.

TAPB combined with general anesthesia can reduce neutrophil count and NLR level in peripheral blood, which may be related to reducing immunosuppression. Different anesthetic drugs have different effects on immunity, recurrence and metastasis. Studies have shown that most intravenous general anesthesia drugs can suppress immune system function and affect cancer progression. Local anesthetics work by blocking voltage-gated sodium channels (VGSC) on nerve cell membranes. VGSCs exist on tumor cell membrane, which is related to tumor cell invasion and metastasis. Local anesthesia can increase NK cell activity, maintain Th1/ balanced Th2 ratio, and reduce plasma cortisol and catecholamine levels during operation. Studies have shown that local anesthesia can reduce stress responses, protect immune function in tumor patients, and reduce opioid use. In addition, levels of vascular endothelial growth factor C (VEGF-C), TGF-β1, acidic fibroblast growth factor, basic fibroblast growth factor, and placental growth factor were reduced in the veins of patients receiving propofol and combined with paravertebral block anesthesia. These growth factors promote angiogenesis and the formation of metastatic tumors, suggesting that anesthesia affects plasma levels of angiogenesis related factors in patients with primary breast cancer, thereby affecting tumor recurrence and metastasis. Compared with general anesthesia alone, general anesthesia combined with epidural anesthesia can improve the clinical survival rate and prognosis. A multicenter randomized controlled clinical study by Short et al reported that, compared with general anesthesia combined with postoperative intravenous analgesia, postoperative epidural analgesia combined with general anesthesia did not reduce the risk of tumor recurrence in patients undergoing abdominal surgery, nor did it extend the tumor free survival of patients.

A meta-analysis of a variety of malignancies has found that high levels of NLR have an impact on the prognosis of patients, especially when NLR> 4.0, NLR is associated with poor overall survival. Perioperative stress, inflammation and immune system suppression can lead to the increase of NLR, and the inhibition of neutrophil apoptosis induced by surgical stress may also play a role. A number of studies have confirmed that the increase of NLR and PLR is independently associated with the prognosis of patients with solid tumors. The study of Wang Yuanchu et al. showed that the preoperative increase of NLR and PLR had a certain value in evaluating the prognosis of ovarian cancer patients, and the same research results were also confirmed in other relevant studies on solid tumors. It has been reported that preoperative high NLR can be an independent factor for postoperative recurrence of gastric cancer. Studies have shown that preoperative NLR>3.4 is an independent risk factor for disease-free survival (DFS) and overall survival (OS), and the DFS and OS in the group with high NLR value are lower than those in the group with low NLR value, respectively. Ultrasound-guided TAPB combined with general anesthesia can not only provide good postoperative analgesia for patients undergoing radical gastrectomy, but also reduce the levels of neutrophils and NLR. Kim et al. 's results combined with NLR suggested that the advantages of TAPB combined with general anesthesia compared with general anesthesia alone were mainly reflected in inhibiting neutrophilism and reducing the influence of neutrophils on tumor microenvironment.

In summary, this project intends to observe whether ultrasound-guided TAPB combined with general anesthesia has any effect on NLR and PLR in peripheral blood of patients undergoing radical endometrial cancer surgery, providing theoretical basis for its clinical use in patients undergoing radical endometrial cancer surgery.

**2 Main research content**

(1) To observe the effects of ultrasound-guided transverse abdominal muscle plane block (TAPB) on neutrophil-lymphocyte ratio (NLR) and platelet-lymphocyte ratio (PLR) in peripheral blood of patients undergoing radical endometrial carcinoma surgery.

Laparoscopic radical resection of endometrial carcinoma was performed under general anesthesia combined with TAPB. The NLR and PLR values of the general anesthesia group alone (GA group) and the general anesthesia combined with TAPB group (GA+TAP group) were compared before and after surgery, 24h and 72h. To investigate the effect of TAPB on peripheral blood NLR and PLR in patients undergoing radical endometrial carcinoma.

(2) To observe the influence of TAPB guided by ultrasound on intraoperative intravenous anesthetic use and postoperative analgesia in patients undergoing radical surgery for endometrial cancer.

Laparoscopic radical resection of endometrial carcinoma was performed under general anesthesia combined with TAPB. Intraoperative anesthetic use and postoperative pain were compared between GA and GA+TAP groups. To explore whether ultrasound-guided TAPB can reduce the use of anesthetic, the occurrence of postoperative nausea and vomiting, the use of postoperative opioids, the improvement of postoperative pain and the reduction of complications in patients undergoing radical surgery for endometrial cancer.

**3 Research methods**

(1) Sample size calculation: According to the pre-experiment results, after 72h, the NLR values of the two groups were 4.80±1.05 and 5.60±1.30 respectively. α=0.05,1-β=0.8, and PASS 15.0 were used to calculate the sample size of about 36 cases in each group. Considering a certain loss of follow-up rate, the estimated sample size was increased by 20%, and about 45 cases were included in each group.

(2) Study subjects: 90 patients who underwent elective radical surgery for endometrial cancer in the First Affiliated Hospital of Shihezi University Medical School from June 7, 2023 to June 1, 2024 were included.

(3) Inclusion criteria: ① Pathological diagnosis of endometrial carcinoma; ② All patients were to undergo laparoscopic radical operation for endometrial carcinoma; ③ American Society of Anesthesiologists (ASA) grades I to III; ④ Age 30~65 years old; ⑤BMI 18~ 30 kg/m2; ⑥ The newly diagnosed patient did not receive any systematic treatment; ⑦ No distant metastasis of other parts occurred; ⑧ The same group of surgeons.

(4) Exclusion criteria: ① skin infection at the puncture site combined with acute and chronic infectious diseases ② other malignant tumors, nervous system diseases, endocrine system dysfunction and blood system diseases; ③ Abnormal coagulation mechanism; (4) Complicated with serious vital organ diseases; ⑤ Other major operations within 3 months before surgery.

**4. Research process**

(1) Ethical review and clinical trial registration: This trial was approved by the Ethics Committee of the First Affiliated Hospital of Shihezi University (KJ2023-015-01) and registered in the Chinese Clinical Trial Registry (ChiCTR2300072186). Written informed consent must be obtained from all participants or their legal representatives prior to the commencement of any proceedings.

(2) Trial grouping: Patients undergoing radical endometrial carcinoma were divided into two groups (50 cases in each group) using random number table method: TAPB+ general anesthesia group (TAP+GA group) and general anesthesia group alone (GA group). Throughout the study, patients, researchers conducting data collection and post-operative follow-up, and clinical staff were unaware of the random assignment.

(3) Test methods: Patients were routinely fasted for 8h and forbidden to drink for 2h before surgery. Peripheral veins were opened after admission to the operating room, and heart rate, heart rhythm, non-invasive blood pressure and SpO2 were routinely monitored. General anesthesia was induced after emergency drugs and equipment were prepared. After nitrogen removal and oxygen administration in the mask, midazolam 0.05 mg/kg, propofol 2.0 mg/kg, sufentanil citrate 0.5 μg/kg and cisatracurium 0.2mg/kg were injected intravenously. After muscle relaxation, oral catheterization was performed under video laryngoscope. After successful intubation, mechanical ventilation was performed, and ventilator parameters were adjusted according to PETCO2 to maintain PETCO2 at 35~45mmHg. Continuous intravenous infusion of propofol at 4-8 mg· kg-1 · h-1, remifentanil at 0.1-0.2 μg· kg-1 · min-1, cisatracurium at 1-2μg· kg-1 · min-1, BIS at 40-60 and muscle relaxation. Perioperative hypotension and bradycardia were treated with 40 μg deoxyadrenaline and 0.5 mg atropine, respectively. In TAP+GA group, the abdominal skin was disinfected after anesthesia induction, and the high-frequency linear array probe of color Doppler ultrasound instrument was used, and the probe was wrapped with a cavity mirror sleeve. The ultrasound probe was placed at the intersection of the midaxillary line of the anterior abdominal wall and the umbilical level to obtain the intraplane ultrasound image of the transverse abdominal muscle, and the puncture needle was guided to the transverse abdominal muscle plane according to the intraplane ultrasound image. When the tip of the needle reached the fascia layer between the internal oblique muscle and the transverse muscle of the abdomen and was withdrawn without blood or air, 2 ml 0.9% sodium chloride injection was injected, and 0.375% ropivacaine 20 ml was injected on both sides of the abdomen after the position was confirmed to be correct. The diffusion of the liquid was observed by ultrasound and complications were determined. TAPB was not performed in GA group alone.

A single intravenous injection of 0.1 μg/kg sufentanil pre-analgesic dose was given at the end of the procedure. All patients were treated with conventional PCIA for postoperative pain management (100 mL normal saline was added with 2 μg/kg sufentanil, 0.06 mg/kg butorphenol tartrate and 8 mg ondansetron, background infusion of 2 mL/h, each 2 mL, interlocking interval of 15 minutes). When the patient's postoperative analgesia is not satisfactory (defined as resting VAS score ≥4), the patient is advised to press the analgesic pump. If the pain does not decrease after two consecutive compressions, the attending physician will administer a single intravenous dose of 1mg butorphanol tartrate for remedial analgesia.

(4) Outcome measures: General data (age, BMI, ASA and clinicopathological stage of the disease) were collected. Operation time, anesthesia time and intraoperative dosage of remifentanil were recorded in the two groups. Peripheral blood neutrophils, platelets and lymphocytes were measured before and 24h and 72h after surgery, and NLR and PLR values were calculated. VAS pain score at 6h, 12h, 24h and 48h, the number of effective pressure of analgesic pump, the first time to get out of bed after surgery, the first time to exhaust gas after surgery, and the occurrence of postoperative nausea and vomiting adverse reactions were recorded in the two groups.

(5) Statistical analysis method: SPSS 25.0 and GraphPad Prism 9.5.1 software were used to analyze and visualize the data. The normality of the measured data is assessed and the mean ± standard deviation (X±SD) is reported for normally distributed data. Independent sample t test was used for comparative analysis. For data with non-normal distribution, the median (quartile range)[M (Q1, Q3)] and Mann-Whitney U test were used for inter-group comparison. Disaggregated data were expressed as percentages or percentages, and chi-square tests were used for comparison between groups. P <0.05 was considered statistically significant.
