# Supplementary material for: Effect of ultrasound-guided transverse abdominal plane block on neutrophil-to-lymphocyte ratio, platelet-to-lymphocyte ratio, and systemic immune inflammation index in patients undergoing radical resection of endometrial carcinoma
Source: PLoS One. 2024 Dec 5;19(12):e0315175. doi: 10.1371/journal.pone.0315175 (PMC11620674; doi:10.1371/journal.pone.0315175)
Supplement: Attachment — Submitted filename: Response to Reviewers.docx [file pone.0315175.s005.docx]

Dear Editor,

Re: Submission of the revised manuscript entitled: “*Effect of ultrasound-guided Transverse Abdominal Plane Block on NLR, PLR, and SII in patients undergoing radical resection of endometrial carcinoma: A randomized controlled trial*” (Submission ID PONE-D-24-32923).

Thank you very much for your letter again and the reviewer’s comments again which we found very helpful. We have carefully followed the comments and revised our manuscript as suggested. Enclosed below are the point-by-point responses to the reviewer’s comments (reviews’ comments in blue, our replies in black). The revised manuscript was marked with red color.

We hope the revised manuscript is satisfactory and acceptable for publication in *PLOS One*. Please let us know if further revisions are needed.

We look forward to your response regarding the revised manuscript.

Best regards,

Yours sincerely

Hong Zhang.

Department of Anesthesiology

First Affiliated Hospital, School of Medical, Shihezi University,

Shihezi 832002, Xinjiang,

P. R. China.

Phone: +86-152-9993-5388

E-mail: 1579927013@qq.com

**Specific responses to the comments:**

First, we appreciate the reviewers’ valuable comments that enabled us to improve the manuscript. In the revised version, we have corrected the mistakes and inaccurate descriptions, as well as revised the entire manuscript, according to the reviewers’ comments. For clear identification, we have changes in red in the “Revised manuscript (marked-up copy)”.

**Response to Reviewer**

Reviewer #1: The authors have responded well to all the statistical concerns raised in the previous review. However, these explanations should be included in the revised manuscript.

Review:

1. The authors should include a brief paragraph in the revised manuscript addressing their responses to the previous critiques regarding sample size estimation (specifically critiques 1 and 2 from reviewer #1).

**Response**: Thank you very much for your earnest and responsible suggestion, we have added the explanation of the sample size estimate in the corresponding section. The specific expression in the manuscript is: It is worth noting that the sample size in the clinical registration scheme is based on previous research experience. After obtaining the ethical review and clinical registration consent, the sample size estimation through the pre-trial can make this study more rigorous. Therefore, the sample size in the clinical registration scheme is slightly inconsistent with the actual sample size. In addition, 72-hour postoperative NLR values were selected for sample size calculation with reference to previous studies. At the same time, SII values can be calculated from the inflammatory indicators that have been collected, so it is ethical to include it in the observation measures. (page 11, line 206-214)

2. In the revised Statistical Analysis section, clearly state: "No multiple comparison adjustments were applied to any of the data analyses."

**Response**: We feel great thanks for your professional review work on our article again. We have added the more rigorous statement you made to the section on statistical methods.(page 12, line 226-227)

Reviewer #2: (No Response)

**Here, I would like to thank the reviewer for your careful review and point out my mistakes and problems. I hope you can give me a chance to publish this research. Here is my sincere gratitude.**
